# Synergistic Strategies in Systemic Therapy for Advanced Hepatocellular Carcinoma

**DOI:** 10.1002/gch2.202500247

**Published:** 2025-12-25

**Authors:** Yongxin Yu, Yulang Jiang, Yipeng Yang, Christian Glandorff, Wenzheng Fang, Mingyu Sun

**Affiliations:** ^1^ People's Hospital Affiliated to Fujian University of Traditional Chinese Medicine Fuzhou China; ^2^ Shanghai University of Traditional Chinese Medicine Shanghai China; ^3^ Shuguang Hospital Affiliated to Shanghai University of Traditional Chinese Medicine Shanghai China; ^4^ University Clinic of Hamburg at the HanseMerkur Center of TCM Hamburg Germany

**Keywords:** hepatocellular carcinoma, immune checkpoint inhibitor, lenvatinib, sorafenib, systemic treatment, tyrosine kinase inhibitor

## Abstract

Hepatocellular carcinoma (HCC) remains the most prevalent primary liver cancer, characterized by alarmingly high mortality rates and low five‐year survival outcomes. A significant challenge in HCC management lies in its advanced‐stage treatment, with most cases identified at advanced, unresectable stages, resulting in poor prognoses and limited treatment options. Over the last decade, considerable advancements have been made in systemic treatment strategies, notably with the introduction of multi‐kinase inhibitors such as sorafenib and lenvatinib, which have redefined the therapeutic landscape for advanced HCC. The emergence of immunotherapy has further revolutionized first‐line treatment, bringing new hope with agents like the PD‐1 inhibitor nivolumab and the CTLA‐4 inhibitor tremelimumab. Moreover, combination regimens such as atezolizumab plus bevacizumab have demonstrated remarkable clinical efficacy, leading to substantial improvements in overall survival and progression‐free survival. Despite the availability of multiple treatment options, clinical trial outcomes remain suboptimal. Key challenges persist in the selection and sequencing of therapies, the development of more diversified combination strategies, and the implementation of downstaging approaches for advanced HCC. This paper aims to provide a comprehensive review of the current progress in systemic therapies for HCC, drawing on extensive research findings and clinical trial data to assess their clinical applications and explore potential challenges. By offering a critical analysis of these therapeutic strategies, this paper seeks to furnish valuable insights and references for ongoing research and future clinical practice, ultimately contributing to improved outcomes in HCC management.

## Graphical abstract

This article comprehensively examines the evolving landscape of systemic therapy for advanced hepatocellular carcinoma. It covers the progress in tyrosine kinase inhibitors and immune checkpoint inhibitors, analyzes the efficacy of novel combination strategies, and discusses current challenges like drug resistance and the lack of biomarkers. Finally, it explores future directions, including sequential therapies and the integration of systemic with local treatments.

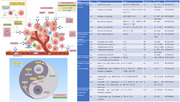

**Abbreviations**
AFP: alpha‐fetoprotein
ASCO: American Society of Clinical Oncology
CTLA‐4: cytotoxic T lymphocyte‐associated antigen 4
DCR: disease control rate
DOR: duration of response
ESMO: European Society for Medical Oncology
HCC: hepatocellular carcinoma
HFSR: hand‐foot skin reaction
ICI: immune checkpoint inhibitor
irAEs: immune‐related adverse events
MDT: multi‐Disciplinary Treatment
mOS: median overall survival
mPFS: median progression‐free survival
ORR: objective response rate
OS: overall survival
PD‐1: programmed cell death 1
PD‐L1: programmed cell death 1 ligand 1
PDO: patient‐derived organoid
PFS: progression‐free survival
RTK: receptor tyrosine kinase
SoC: standard of care
TIME: tumor immune microenvironment
TKD: tyrosine kinase domain
TKI: tyrosine kinase inhibitor
TME: tumor microenvironment
TRAE: treatment‐related adverse events

## 1 Introduction

In recent years, hepatocellular carcinoma (HCC) has emerged as one of the most prevalent malignant tumors worldwide, with its incidence and mortality rates steadily increasing, posing a severe public health challenge [1]. The development and progression of HCC involve various factors [2], including chronic liver disease, viral hepatitis, alcoholic liver disease, and non‐alcoholic fatty liver disease [3]. Due to the subtlety of early symptoms, many patients are diagnosed at mid‐to‐late stages, leading to poor prognosis [4]. With rapid advancements in molecular biology, immunology, and drug development [5, 6], systemic treatment strategies for HCC have achieved remarkable progress. These strategies encompass molecular targeted therapy, immunotherapy, and chemotherapy, with molecular targeted therapy and immunotherapy showing substantial potential in HCC treatment [7].

Molecular targeted therapy inhibits the growth and spread of cancer cells by disrupting specific molecular pathways, delaying tumor progression by inhibiting angiogenesis and cell proliferation signaling pathways [8]. However, issues such as drug resistance and side effects remain significant challenges, prompting ongoing exploration of new targets and combination therapies [9]. Immunotherapy, particularly immune checkpoint inhibitors (ICIs) like PD‐1/PD‐L1 inhibitors, has significantly improved survival rates for some patients, enhancing the body's anti‐tumor immune response by lifting immune suppression on tumor cells [10]. Nevertheless, the effectiveness of immunotherapy varies greatly among individuals, making the prediction and monitoring of treatment response, as well as the management of immune‐related adverse events, key areas of clinical research [11].

Overall, systemic treatment strategies for HCC are continually evolving and improving, and Multi‐Disciplinary Treatment (MDT) is becoming increasingly prevalent [12]. Future research must further elucidate the mechanisms of HCC development [13], explore new therapeutic targets and biomarkers [14], and employ precision medicine to achieve individualized treatment, ultimately improving patient survival and quality of life [15].

## 2 Systemic Treatment Strategies for HCC

With the deepening research on HCC and the continuous advancement of clinical technology, the treatment strategies for HCC have gradually evolved from single surgical therapy to diversified and systematic integrated treatment plans [16]. Statistics show that 50%–60% of HCC patients are diagnosed in the late stages of the disease or progress after surgery or local treatment, requiring systemic therapy. The importance of systemic therapy in the treatment of HCC cannot be overstated [17]. As a complex systemic disease, the development of HCC is often accompanied by metabolic, immune, and tumor microenvironment (TME) changes in the body [18]. These changes not only affect the progression and metastasis of HCC but also influence the patient's response to treatment and prognosis. Therefore, relying solely on local surgical resection, radiotherapy, or interventional therapy often fails to achieve ideal treatment outcomes.

**FIGURE 1:**
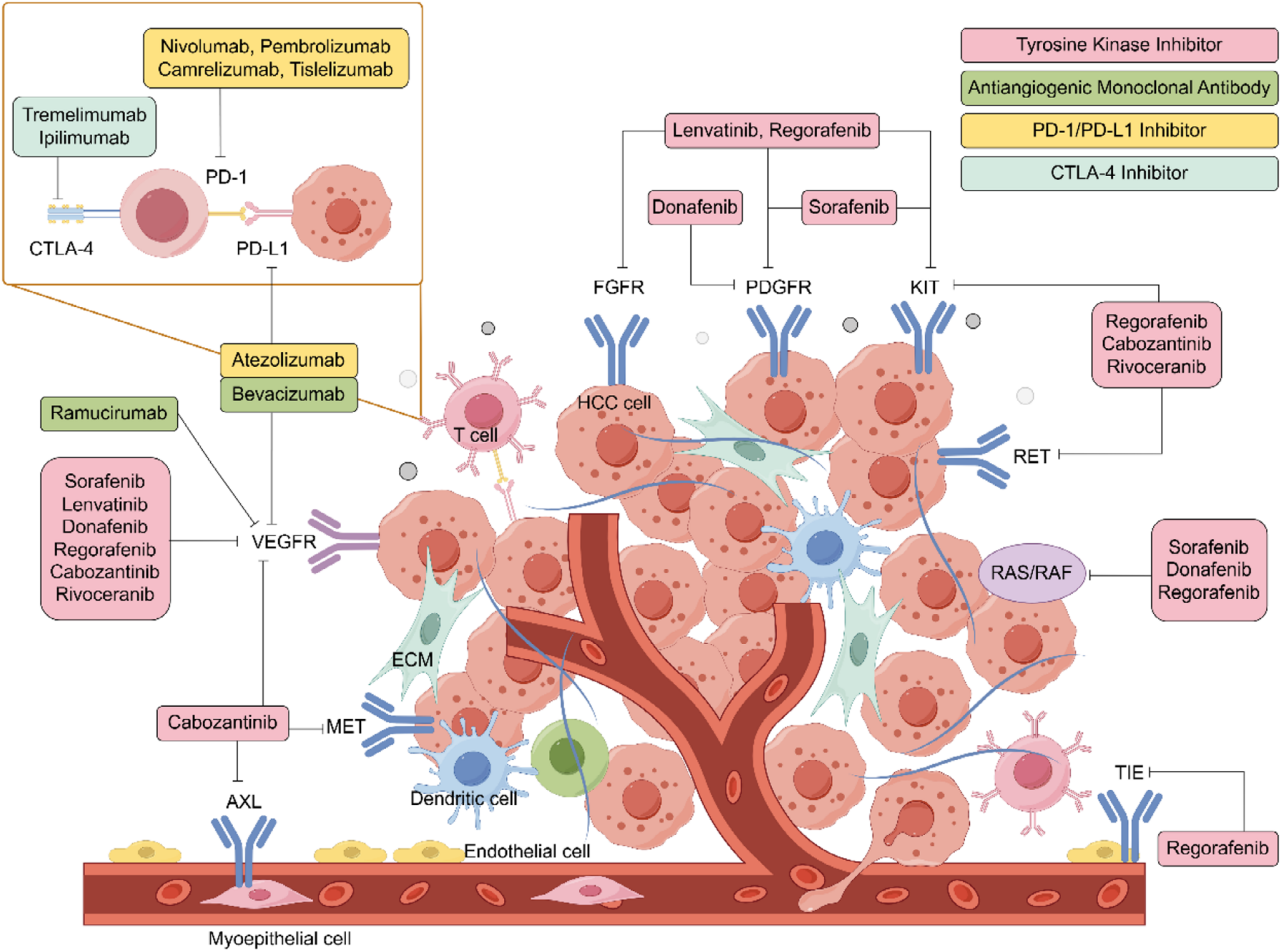
Overview of systemic therapy for hepatocellular carcinoma. Systemic therapy encompasses targeted therapy and immunotherapy. Targeted therapy primarily involves tyrosine kinase inhibitors (red box) and antiangiogenic monoclonal antibodies (green box), while immunotherapy mainly includes PD‐1/PD‐L1 inhibitors (yellow box) and CTLA‐4 inhibitors (viridescence box). Tyrosine kinase inhibitors exert their antitumor effects by inhibiting various receptor tyrosine kinases in tumor cells, such as VEGFR, FGFR, MET, KIT, RET, and TIE, among others. Antiangiogenic monoclonal antibodies primarily inhibit tumor angiogenesis via VEGFR. PD‐1/PD‐L1 inhibitors enhance the antitumor activity of T cells and prevent immune escape of tumor cells by blocking the PD‐1 receptor on T cells or the PD‐L1 receptor on tumor cells. CTLA‐4 inhibitors restore T cell antitumor immunity by inhibiting the interaction between CTLA‐4 and CD80/CD86.

Since 2007, when the first systemic treatment drug for HCC, sorafenib, was approved for treating advanced HCC, patients in desperate situations have found new treatment options [19]. Since then, various single‐drug treatment regimens have emerged, sparking a wave of clinical trials for advanced HCC worldwide. However, the results have been less than satisfactory, with only sorafenib and lenvatinib currently being the main first‐line TKI drugs [20]. The rise of targeted combined immunotherapy after 2020 marks the advent of a new era in systemic therapy [21]. The development of systemic therapeutic drugs has profoundly changed the management landscape of HCC (refer to Figure 1).

### 2.1 Targeted Therapy

Targeted therapy is presently a hot topic in the non‐surgical treatment of HCC. Sorafenib's good efficacy and prolonged survival in advanced HCC have established the value and importance of targeted therapy in advanced HCC [8]. Through the unremitting efforts of researchers, the field of targeted therapy has blossomed. Currently, there are mainly two types of targeted therapy drugs: tyrosine kinase inhibitors (TKIs) and antiangiogenic monoclonal antibodies, as detailed in Table 1.

**TABLE 1:** The list of targeted therapeutic drugs.

Type	Line	Clinical trial name and number	Drug	Target	Sample size	mOS (months)	mPFS (months)	ORR (%)	DCR (%)	≥Grade 3 TRAEs (%)	Regulatory approval
Tyrosine kinase inhibitor	1st	Oriental, NCT00492752	Sorafenib (400 mg) or placebo orally twice daily in 6‐week cycles	VEGFR2‐3, PDGFR‐β, RAF	*n* = 150 for sorafenib vs *n* = 76 for placebo	6.5 vs 4.2	2.8 vs 1.4	/	47.3	20.1	FDA (2007), EMA (2007), NMPA (2008)
	1st	REFLECT, NCT01761266	Lenvatinib: patients weighing <60 kg received 8 mg/day; patients weighing ≥60 kg received 12 mg/day; sorafenib (400 mg) orally twice daily	VEGFR1‐3, FGFR1‐4, PDGFR‐α, RET, KIT	*n* = 468 for lenvatinib vs *n* = 463 for sorafenib	13.6 vs 12.3	7.4 vs 3.7	18.8	/	/	FDA (2018), EMA (2018), NMPA (2018)
	1st	ZGDH3, NCT02645981	Donafenib (0.2 g) or sorafenib (0.4 g) orally twice daily	VEGFR, PDGFR, RAF	*n* = 328 for donafenib vs *n* = 331 for sorafenib	12.1 vs 10.3	3.7 vs 3.6	4.6	30.8	38	NMPA (2021)
	2nd	RESORCE, NCT01774344	Regorafenib 160 mg or placebo orally once daily during weeks 1–3 of each 4‐week cycle	VEGFR1‐3, TIE2, PDGFR‐β, FGFR, RAF, KIT, RET	*n* = 374 for regorafenib vs n = 193 for placebo	10.6 vs 7.8	/	/	/	52	FDA (2017), EMA (2017), NMPA (2017)
	2nd	CELESTIAL, NCT01908426	Cabozantinib (60 mg once daily) or placebo orally	VEGFR1‐3, KIT, RET, MET, AXL	*n* = 470 for cabozantinib vs *n* = 237 for placebo	10.2 vs 8.0	5.2 vs 1.9	4	64	68	FDA (2019), EMA (2019)
	2nd	AHELP, NCT02329860	Rivoceranib 750 mg or placebo orally once daily	VEGFR2, KIT, RET	*n* = 267 for rivoceranib vs *n* = 133 for placebo	8.7 vs 6.8	/	10.7	/	27.6	NMPA (2020)
Antiangiogenic monoclonal antibody	2nd	REACH‐2, NCT02435433	Ramucirumab 8 mg/kg intravenously every 2 weeks or placebo	VEGFR2	*n* = 197 for ramucirumab vs *n* = 95 for placebo	8.5 vs 7.3	2.8 vs 1.6	5	/	/	FDA (2019), EMA (2019)

#### 2.1.1 Tyrosine Kinase Inhibitors

Receptor tyrosine kinases (RTKs) are a group of transmembrane receptors composed of 20 subtypes (Table 2), playing important roles in cell survival, proliferation, differentiation, metabolism, regeneration, and apoptosis pathways [22]. The enzymatic activity of RTKs in normal cells is relatively stable, and the cascade reactions of biological signals are strictly controlled. However, due to autocrine or paracrine stimulation, mutations, or overexpression, the affinity between RTK receptors and their ligands increases, leading to increased activity of the tyrosine kinase domain (TKD), which in turn leads to oncogenic activation, tumor metastasis, angiogenesis, and therapeutic resistance [23]. Therefore, many researchers believe that tyrosine kinase proteins are key mediators in cancer development, making the development of corresponding RTK‐targeted drugs imperative [24]. Over the past decade, many TKI products have been proven to improve the therapeutic effects for HCC patients, with more targeting mechanisms gradually being unveiled.

**TABLE 2:** The classification of RTK subfamilies.

Class	Subfamily name	Members
1	ErbB	EGFR (ErbB1, HER1), ErbB2 (HER2), ErbB3 (HER3), ErbB4 (HER4)
2	Insulin R	INSR (IR), IGFR, InsRR (IRR)
3	NGFR	TrkA, TrkB, TrkC
4	FGFR	FGFR1, FGFR2, FGFR3, FGFR4
5	VEGFR	VEGFR1 (Flt1), VEGFR2 (KDR, Flk1), VEGFR3 (Flt4)
6	PDGFR	PDGFRα, PDGFRβ, CSFR (Fms), FLT3 (Flk2), KIT (SCFR)
7	TAM	TYRO3, AXL (UFO), MER (MERTK)
8	DDR	DDR1, DDR2
9	Eph	EphA1‐8, EphA10, EphB1‐4, EphB6
10	TIE	TIE1, TIE2 (TEK)
11	HGFR	MET (HGFR), Ron (MSPR, MST1R)
12	PTK7	PTK7 (CCK4)
13	RET	RET
14	ALK	ALK, LTK
15	MuSK	MuSK
16	ROR	ROR1, ROR2
17	RYK	RYK
18	ROS	ROS
19	STYK1	STYK1 (NOK, RTK106)
20	LMTK	LMTK1 (LTK1, AATYK1), LMTK2 (LTK2, AATYK2, BREK), LMTK3 (LTK3, AATYK3)

##### 2.1.1.1 Sorafenib

Sorafenib was the first TKI product used to treat advanced HCC. Sorafenib can inhibit RAF activation and downstream MEK/ERK phosphorylation by targeting and antagonizing PDGFR‐β, VEGFR2, and VEGFR3, leading to obstruction of gene transcription involved in angiogenesis, thereby reducing tumor angiogenesis and energy supply [25]. Additionally, inhibiting MEK/ERK phosphorylation can downregulate Cyclin D1 levels, causing cell cycle arrest from G0 to G1 phase, ultimately inhibiting tumor cell growth and proliferation [26]. Moreover, sorafenib can promote tumor cell apoptosis by inhibiting eIF4E phosphorylation and the expression of anti‐apoptotic factor Mcl‐1 [27]. The SHARP (NCT00105443) and Oriental (NCT00492752) global phase III randomized controlled trials have shown that sorafenib significantly improves overall survival (OS) in patients with unresectable and advanced HCC [28, 29]. However, with increasing clinical usage, sorafenib resistance has become more prominent [30], making the exploration of combination applications or new targeted therapeutic drugs a key solution.

##### 2.1.1.2 Lenvatinib

Lenvatinib is a type of RTK inhibitor that can inhibit the expression and activity of VEGFR1‐3, FGFR1‐4, PDGFR, RET, and KIT [31]. Compared to sorafenib, lenvatinib can more effectively inhibit VEGFR and FGFR, significantly suppressing the phosphorylation of FGFR1‐4 substrates and reducing ERK1/2 phosphorylation, leading to better inhibition of angiogenesis [32]. In some FGFR4‐expressing HCC cell lines resistant to sorafenib, lenvatinib can inhibit the FGFR4/ERK signaling pathway, overcoming sorafenib resistance, and inhibiting HCC cell proliferation [33]. A phase II study (NCT00946153) found that lenvatinib has good clinical efficacy and safety for unresectable HCC [34]. The REFLECT trial (NCT01761266), a multicenter randomized non‐inferiority phase III trial, confirmed that lenvatinib is non‐inferior to sorafenib in terms of median overall survival (mOS) demonstrating non‐inferiority (13.6 months vs. 12.3 months) and showing better progression‐free survival (PFS) and objective response rate (ORR) [35].

##### 2.1.1.3 Donafenib

Donafenib is a deuterated derivative of sorafenib that targets and antagonizes VEGFR, PDGFR, and RAF kinases, blocking tumor angiogenesis and proliferation. It has been approved in China as a first‐line treatment for patients with unresectable advanced HCC [36]. A randomized, multicenter, open, parallel‐controlled phase II‐III clinical study (ZGDH3, NCT02645981) compared donafenib with sorafenib, finding that the mOS in the donafenib group was significantly improved compared to the control group (12.1 months vs. 10.3 months), with a 17% reduction in the risk of death. The incidence of severe adverse reactions (TRAE) was also significantly lower in the trial group (38% vs. 50%) [37]. Therefore, donafenib may be a highly promising first‐line treatment drug.

##### 2.1.1.4 Regorafenib

Regorafenib is a multi‐kinase inhibitor structurally similar to sorafenib, with the addition of a fluorine atom at a key site, giving it better bioactivity than sorafenib. It targets angiogenesis (VEGFR1‐3, TIE2), tumor stroma (PDGFR‐β, FGFR), and receptor tyrosine kinases (KIT, RET, and RAF) [38]. In 2017, regorafenib was approved for the treatment of advanced HCC patients who had failed sorafenib treatment [39]. The phase III clinical trial RESORCE (NCT01774344), which compared regorafenib and placebo as second‐line treatments after sorafenib failure, found that the OS in the regorafenib group was significantly longer (10.6 months vs. 7.8 months), and the ORR was significantly higher (10.6% vs. 4.1%). The safety profile of regorafenib was comparable to the control group [40]. However, clinical research data on regorafenib are relatively limited, with only about 30% of patients benefiting from the treatment. More trial data is needed to support its use or to explore new combination strategies [41].

##### 2.1.1.5 Cabozantinib

Cabozantinib is a multi‐tyrosine kinase inhibitor with primary targets including VEGFR1‐3, KIT, RET, MET, and AXL [42]. The phase III clinical trial CELESTIAL (NCT01908426) showed that patients in the cabozantinib group had significantly longer mOS (10.2 months vs. 8.0 months) compared to the placebo group. The median progression‐free survival (mPFS) was 5.2 months vs. 1.9 months, and the ORR was 4% vs. 1%. However, the rate of high‐grade TRAE in the cabozantinib group was twice that of the placebo group, including liver failure, portal vein thrombosis, hepatorenal syndrome, and pulmonary embolism [43]. Additionally, real‐world studies indicate that cabozantinib treatment incurs substantial economic costs, with medical expenses rising as treatment progresses [44]. Therefore, it may not be a cost‐effective option for second‐line HCC therapy.

##### 2.1.1.6 Rivoceranib

Rivoceranib is a small‐molecule TKI independently developed in China, highly selective for VEGFR2. It inhibits the proliferation and migration of endothelial cells by binding to VEGFR2, thereby inhibiting tumor angiogenesis and inducing tumor cell apoptosis [45]. The phase III clinical trial AHELP (NCT02329860), a randomized, multicenter, double‐blind, placebo‐controlled study, showed that rivoceranib significantly prolonged mOS (8.7 months vs. 6.8 months), increased ORR (10.7% vs. 1.5%), reduced the risk of death by 21.5%, and decreased the risk of disease progression by 52.9%. Common TRAEs included hypertension, hand‐foot skin reaction (HFSR), and thrombocytopenia [46]. Currently, rivoceranib is used as a second‐line treatment for HCC patients who have failed first‐line systemic anti‐tumor therapy, with ongoing international clinical trials exploring combination therapies [47].

#### 2.1.2 Antiangiogenic Monoclonal Antibody

HCC cells have enhanced angiogenic capabilities, promoting capillary dilation, basement membrane disruption, extracellular matrix remodeling, pericyte shedding, and endothelial cell differentiation through the continuous release or upregulation of various pro‐angiogenic factors. This maintains a highly active phase of angiogenesis, increasing blood circulation to meet the tumor's oxygen and nutritional needs and alleviating metabolic stress [48]. Traditional anti‐angiogenesis therapies are based on “starving tumors” by blocking angiogenesis to induce tumor cell death, thereby obstructing the energy supply to tumor tissues. However, clinical benefits are often not long‐lasting, with high recurrence and resistance [49]. Hence, new antiangiogenic monoclonal antibodies have been developed, offering high purity, sensitivity, specificity, and minimal cross‐reactivity. These antibodies combat tumors by normalizing tumor vasculature, alleviating hypoxia in the TME, increasing drug concentration in tissues, and limiting distant invasion and metastasis [50]. Researchers have proposed various combination strategies with antiangiogenic therapies to maximize their benefits and enhance the efficacy of chemotherapy, radiotherapy, and immunotherapy.

##### 2.1.2.1 Ramucirumab

Ramucirumab is the first humanized monoclonal antibody that specifically targets VEGFR2, effectively inhibiting intratumoral angiogenesis. The phase III REACH trial (NCT01140347) evaluated ramucirumab as a second‐line treatment for patients intolerant to or failing sorafenib. The results showed that ramucirumab did not meet its primary endpoint, failing to significantly improve mOS in advanced HCC patients. However, subgroup analysis revealed that patients with baseline alpha‐fetoprotein (AFP) ≥400 ng/mL significantly benefited from ramucirumab, with mOS extended to 7.8 months vs. 4.2 months, while no significant benefit was observed in patients with baseline AFP <400 ng/mL [51]. The subsequent REACH‐2 trial (NCT02435433) confirmed the efficacy of ramucirumab in advanced HCC patients with baseline AFP ≥400 ng/mL [52]. Consequently, ramucirumab has been approved by the US FDA as the preferred second‐line targeted therapy for advanced HCC patients with baseline AFP ≥400 ng/mL [53].

##### 2.1.2.2 Bevacizumab

Bevacizumab, the earliest approved angiogenesis inhibitor, is a macro‐molecular recombinant human monoclonal antibody that inhibits tumor angiogenesis by neutralizing all VEGFR subtypes and blocking VEGF pathway signaling, thereby inhibiting tumor growth [54]. First approved by the FDA in 2004 for the treatment of metastatic colorectal cancer, bevacizumab has since been expanded to include multiple cancer indications, yielding satisfactory results in numerous clinical trials. As a result of its excellent anti‐angiogenic activity, bevacizumab is often used in various combination treatment strategies, including for HCC [55].

### 2.2 Immune Checkpoint Inhibitors

The development and progression of tumors depend not only on the characteristics of the tumor cells themselves but also on the tumor immune microenvironment (TIME). In the TME, interactions between cancer cells, immune cells, and immune molecules allow cancer cells to escape immune surveillance, leading to their continued proliferation, angiogenesis, and metastasis [56]. Programmed cell death 1 (PD‐1), programmed cell death 1 ligand 1 (PD‐L1), and cytotoxic T lymphocyte‐associated antigen 4 (CTLA‐4) are classical immune checkpoints. Over the past decade, ICIs (such as PD‐1/PD‐L1 and CTLA‐4 monoclonal antibodies) have made significant progress in the treatment of advanced HCC [57], as shown in Table 3.

**TABLE 3:** The list of immune checkpoint inhibitors.

Type	Line	Clinical trial name and number	Drug	Target	Sample size	mOS (months)	mPFS (months)	ORR (%)	DCR (%)	≥Grade 3 TRAEs (%)	Regulatory approval
PD‐1/PD‐L1 inhibitor	2nd	CheckMate 459, NCT02576509	Nivolumab (240 mg intravenously every 2 weeks) or sorafenib (400 mg orally twice daily)	PD‐1	*n* = 371 for nivolumab vs *n* = 372 for sorafenib	16.4 vs 14.7	3.7 vs 3.8	15.4	/	12	/
	2nd	KEYNOTE‐240, NCT03062358	Pembrolizumab 200 mg or placebo intravenously every 3 weeks for at least 35 cycles	PD‐1	*n* = 278 for pembrolizumab vs *n* = 135 for placebo	13.9 vs 10.6	3.0 vs 2.8	18.3	/	52.7	/
	2nd	/, NCT02989922	Camrelizumab 3 mg/kg intravenously every 2 or 3 weeks	PD‐1	*n* = 109 for 2 week vs *n* = 108 for 3 week	/	/	14.7	/	22	NMPA (2020)
	2nd	RATIONALE‐301, NCT03412773	Tislelizumab (200 mg intravenously every 3 weeks) or sorafenib (400 mg orally twice daily)	PD‐1	*n* = 342 for tislelizumab vs *n* = 332 for sorafenib	15.9 vs 14.1	2.2 vs 3.6	14.3	/	48.2	NMPA (2021)
	1st	HIMALAYA, NCT03298451	Durvalumab (1500 mg intravenously once every 4 weeks) or sorafenib (400 mg orally twice daily)	PD‐L1	*n* = 389 for durvalumab vs *n* = 389 for sorafenib	/	/	/	/	/	/
CTLA‐4 inhibitor	2nd	Study22, NCT02519348	T300+D (tremelimumab 300 mg plus durvalumab 1,500 mg [one dose each during the first cycle]), durvalumab (1500 mg once every 4 weeks), tremelimumab (750 mg once every 4 weeks), T75+D (tremelimumab 75 mg once every 4 weeks plus durvalumab 1,500 mg once every 4 weeks [four doses]	CTLA‐4	*n* = 75 for T300+D vs *n* = 104 for durvalumab vs *n* = 69 for tremelimumab vs *n* = 84 for T75+D	18.7 vs 13.6 vs 15.1 vs 11.3	/	24	/	37.8	/

#### 2.2.1 PD‐1/PD‐L1 Inhibitors

PD‐1 is a transmembrane protein expressed on different types of immune cells, primarily CD8+ T cells. It plays a crucial role in inhibiting immune responses and promoting self‐tolerance by regulating T cell activity, activating antigen‐specific T cell apoptosis, and inhibiting Treg apoptosis. This serves as a normal homeostatic mechanism of the immune system [58]. In the TME, hypoxia and inflammation induce high expression of PD‐1 on infiltrating T cells, while tumor cells express high levels of PD‐1's ligand, PD‐L1. This leads to sustained activation of the PD‐1 pathway, suppressing T cell function and preventing them from killing tumor cells [59]. PD‐1 inhibitors can block the highly expressed PD‐1 protein on immune cells, partially restoring T cell cytotoxic function. PD‐L1 inhibitors can antagonize the highly expressed PD‐L1 molecules on tumor cells, eliminating the suppression of T cells by the tumor. Both inhibitors can block the PD‐1/PD‐L1 pathway, enhancing immune function, improving T cell recognition of tumor cells, and activating their cytotoxic effects.

##### 2.2.1.1 Nivolumab

Nivolumab is a high‐selectivity, fully humanized monoclonal IgG4 antibody targeting the PD‐1 receptor. In an open‐label, non‐comparative, dose‐escalation and expansion phase I/II trial (CheckMate‐040, NCT01658878), the efficacy and safety of nivolumab were evaluated in patients with HCC who had failed sorafenib treatment. The ORR reached 15%, and the disease control rate (DCR) was 58%, demonstrating good antitumor activity [60]. In 2017, the US FDA approved nivolumab for the treatment of HCC patients who had progressed on sorafenib. However, in a randomized, multicenter phase III trial (CheckMate‐459, NCT02576509), results showed that the ORR in the nivolumab group was higher than that in the sorafenib group (57% vs. 26%), but there was no significant benefit in mOS (16.4 months vs. 14.7 months) [61]. These clinical trials indicated a noticeable trend toward improved mOS in patients receiving nivolumab or sorafenib, but they did not show that immunotherapy was superior to sorafenib. More optimized treatment strategies, such as combination therapy, may be needed to explore the value of nivolumab in HCC [62].

##### 2.2.1.2 Pembrolizumab

Pembrolizumab is a humanized monoclonal IgG4‐kappa antibody targeting the PD‐1 receptor. In the phase II trial KEYNOTE‐224 (NCT02702414), pembrolizumab was used as second‐line treatment for patients with advanced HCC who had progressed after sorafenib treatment. Results showed that the ORR for the pembrolizumab group was 17%, the mPFS was 4.9 months, and the mOS was 13.2 months [63]. However, in the subsequent phase III trial KEYNOTE‐240 (NCT02702401), pembrolizumab as a second‐line treatment for advanced HCC did not reach the predefined statistical significance for OS and PFS [64]. In a phase III trial conducted in China (KEYNOTE‐394, NCT03062358), results showed that pembrolizumab significantly extended mOS (14.6 months vs. 13.0 months), reduced the risk of death by 21%, and more than doubled the 3‐year OS rate compared to the placebo group (23.4% vs. 11.0%). This demonstrated that pembrolizumab is an indispensable choice for second‐line standard treatment of HCC and is a relatively safe treatment option for patients who are intolerant to targeted therapies or at risk of bleeding [65].

##### 2.2.1.3 Camrelizumab

Camrelizumab is an IgG4‐kappa humanized monoclonal antibody developed in China that targets the PD‐1 receptor. Generally, the lower the IC50 and EC50 values of PD‐1 inhibitors, the higher their affinity for PD‐1. Camrelizumab has an IC50 value of 0.7 nmol/L and an EC50 value of 0.38 nmol/L, which are similar to those of pembrolizumab, indicating that camrelizumab has high affinity for PD‐1 and strong antitumor activity [66]. A phase II clinical trial (NCT02989922) showed that camrelizumab as a second‐line treatment had an ORR of 14.7%, a 6‐month overall survival rate of 74.4%, and a mOS of 13.8 months [67]. These results confirm that camrelizumab has good efficacy and safety in the treatment of advanced HCC, making it a valuable treatment option.

##### 2.2.1.4 Tislelizumab

Tislelizumab, another PD‐1 receptor inhibitor developed in China, is a humanized IgG4 monoclonal antibody. It was specifically designed with Fc segment modifications to remove FcγRI binding, effectively avoiding the depletion of effector T cells and adverse reactions caused by macrophages [68]. A phase II clinical trial RATIONALE‐208 (NCT03419897) showed that tislelizumab as a second‐line treatment had an ORR of 13.3%. Subsequently, a phase III clinical trial RATIONALE‐301 (NCT03412773) demonstrated that compared to sorafenib, tislelizumab had a higher ORR and more durable duration of response (DOR) with no significant difference in OS, and it exhibited better safety [69]. These findings suggest that tislelizumab represents a potential first‐line treatment option for patients with unresectable HCC.

##### 2.2.1.5 Durvalumab

Durvalumab is a high‐affinity humanized IgG1 monoclonal antibody targeting the PD‐L1 receptor. It can directly bind to PD‐L1 receptors on tumor cells, blocking the interaction between PD‐L1 and PD‐1/CD80 on T cells, thereby inhibiting tumor immune escape and activating T cell antitumor activity [70]. A phase II clinical trial Study 22 (NCT02519348) evaluated the efficacy and safety of durvalumab monotherapy. The incidence of ≥ grade 3 immune‐related adverse events (irAEs) was 20.8%, showing the lowest rate of adverse events and a relatively good ORR of 10.6%, with an OS of 13.6 months. Although durvalumab monotherapy demonstrated some clinical activity, combination therapies appear to be more effective and warrant further research [71].

#### 2.2.2 CTLA‐4 Inhibitor

CTLA‐4, also known as CD152, is a transmembrane receptor on T cells that shares the B7 molecule ligands with CD28. It primarily derives from glycoproteins expressed by CD4+ and CD8+ T cells and acts as an immune checkpoint, downregulating immune responses [72]. CTLA‐4 binds to CD80 (B7‐1) and CD86 (B7‐2) on antigen‐presenting cells, inducing T cell anergy and negatively regulating immune responses [73]. Additionally, CTLA‐4 is expressed on Treg cells, enhancing their activity and differentiation to suppress T cell activation, mediating tumor immune escape, and promoting cancer progression [74]. Therefore, CTLA‐4 inhibitors can restore T cell antitumor immunity by inhibiting CTLA‐4 interaction with CD80/CD86, thereby inhibiting tumor progression.

##### 2.2.2.1 Tremelimumab

Tremelimumab is a fully human IgG2 monoclonal antibody targeting the CTLA‐4 receptor, relieving CTLA‐4‐mediated T cell immune suppression, and was the first ICI drug used to treat advanced HCC [75]. As early as 2013, a phase II clinical trial (NCT01008358) evaluated the efficacy and safety of tremelimumab in HCC caused by chronic hepatitis C, with an ORR of 17.6% and a PFS of 6.48 months, indicating some antitumor potential [76]. However, in the Study 22 clinical trial (NCT02519348), 43.5% of patients in the tremelimumab monotherapy group experienced grade 3 or higher irAEs, including common immune‐related adverse events [71]. As a result, researchers began exploring combination therapies involving tremelimumab, such as the STRIDE regimen [77], or other combinations like tremelimumab with radiation therapy (jRCT2031210046), TACE (NCT05301842), and ablation therapy (NCT01853618) [78, 79].

##### 2.2.2.2 Ipilimumab

Ipilimumab is a fully human IgG1κ monoclonal antibody that blocks the CTLA‐4 inhibitory receptor on T cells. Ipilimumab can restore T cell cytotoxicity against tumor cells, primarily used in combination therapies for HCC [80]. Studies have shown that ipilimumab alone can enhance IL‐2 production, and when combined with nivolumab, it can stimulate higher IL‐2 release in peripheral mononuclear cells and synergistically enhance effector and memory T cell responses [81].

### 2.3 Combination Therapy

**FIGURE 2:**
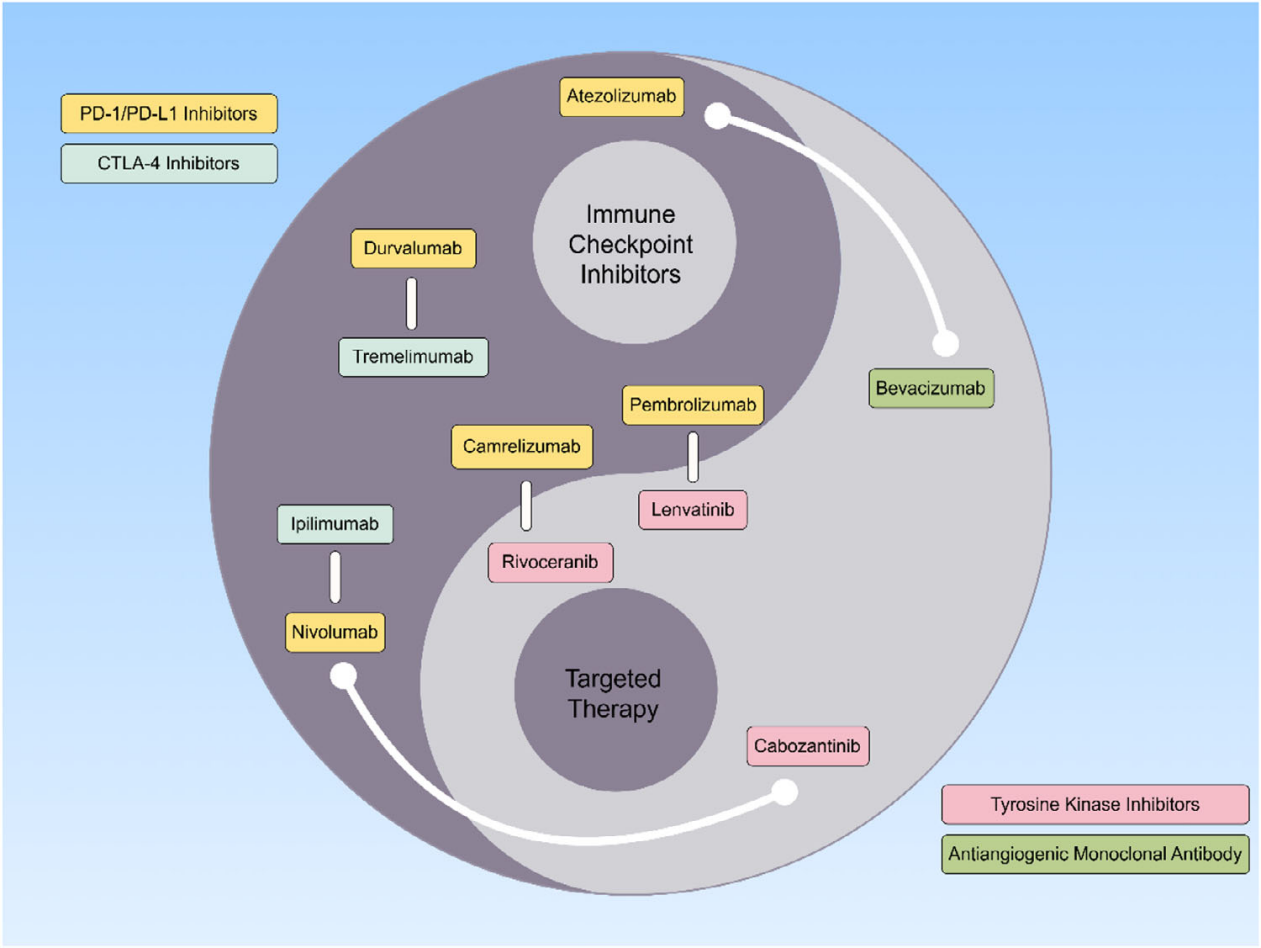
Schematic diagram of combination therapy for hepatocellular carcinoma. Combination therapy primarily encompasses targeted therapy plus immunotherapy and Dual immunotherapy. In the illustration, targeted therapy includes tyrosine kinase inhibitors (red box) and antiangiogenic monoclonal antibodies (green box). Immunotherapy consists of PD‐1/PD‐L1 inhibitors (yellow box) and CTLA‐4 inhibitors (viridescence box). Targeted therapy plus immunotherapy involves regimens such as atezolizumab plus bevacizumab, pembrolizumab plus lenvatinib, camrelizumab plus rivoceranib, nivolumab plus cabozantinib. Dual immunotherapy primarily includes nivolumab plus ipilimumab, tremelimumab plus durvalumab.

While targeted and immunotherapy drugs can improve the prognosis of patients with advanced HCC to a certain extent, the complex TME of HCC limits the efficacy of monotherapy [18]. Combination therapies involving targeted and immunotherapies can act on multiple targets simultaneously, enhancing the body's antitumor effects. Available research data show that combination immunotherapies for advanced HCC are more effective than various monotherapies [8]. Current combination therapy strategies mainly include immunotherapy combined with targeted therapy, dual immunotherapy, and combinations of targeted or immunotherapy with local treatments (refer to Figure 2), as detailed in Table 4.

**TABLE 4:** The list of combination strategies.

Type	Line	Clinical trial name and number	Drug	Target	Sample size	mOS (months)	mPFS (months)	ORR (%)	DCR (%)	≥Grade 3 TRAEs (%)	Regulatory approval
Targeted therapy plus immunotherapy	1st	IMbrave150, NCT03434379	Atezolizumab 1200 mg plus bevacizumab 15 mg per kilogram body weight intravenously every 3 weeks or sorafenib 400 mg orally twice daily	PD‐L1, VEGFR	*n* = 336 for atezolizumab plus bevacizumab vs *n* = 165 for sorafenib	19.2 vs 13.4	6.9 vs 4.3	29.8	/	56.5	FDA (2020), EMA (2020), NMPA (2021)
	1st	LEAP‐002, NCT03713593	Lenvatinib 8 mg (body weight <60 kg) or 12 mg (body weight ≥60 kg) oral once daily plus pembrolizumab 200 mg intravenous or placebo every 3 weeks for up to 35 cycles	PD‐1, VEGFR1‐3, FGFR1‐4, PDGFR‐α, RET, KIT	*n* = 395 for lenvatinib plus atezolizumab vs *n* = 399 for lenvatinib plus placebo	21.2 vs 19.0	8.2 vs 8.0	26.1	/	30	/
	1st	CARES‐310, NCT03764293	Camrelizumab 200 mg intravenously every 2 weeks plus rivoceranib 250 mg orally once daily or sorafenib 400 mg orally twice daily	PD‐1, VEGFR2, KIT, RET	*n* = 272 for camrelizumab plus rivoceranib vs *n* = 271 for sorafenib	22.1 vs 15.2	5.6 vs 3.7	25.4	/	38	NMPA (2023)
	2nd	CheckMate 040, NCT01658878	Nivolumab 240 mg once every 2 weeks plus cabozantinib 40 mg once daily (doublet arm) or nivolumab 3 mg/kg every 2 weeks plus cabozantinib 40 mg once daily with ipilimumab 1 mg/kg once every 6 weeks (triplet arm)	PD‐1, VEGFR1‐3, KIT, RET, MET, AXL, CTLA‐4	*n* = 36 for doublet arm vs *n* = 35 for triplet arm	20.2 vs 22.1	5.1 vs 4.3	17	/	50	/
Dual immunotherapy	1st	CheckMate 9DW, NCT04039607	Nivolumab (1 mg/kg) plus ipilimumab (3 mg/kg) intravenously every 3 weeks for up to four doses, followed by nivolumab 480 mg every 4 weeks or investigator's choice of either oral lenvatinib (8 mg or 12 mg mg daily depending on bodyweight) or oral sorafenib (400 mg twice daily)	PD‐1, CTLA‐4	*n* = 335 for nivolumab plus ipilimumab vs *n* = 333 for lenvatinib or sorafenib	23.7 vs 20.6	/	/	/	41	/
	1st	HIMALAYA, NCT03298451	Tremelimumab 300 mg for one dose plus durvalumab 1500 mg once every 4 weeks; sorafenib 400 mg twice daily	CTLA‐4, PD‐L1	*n* = 393 for STRIDE vs *n* = 389 for sorafenib	16.4 vs 13.8	3.8 vs 4.1	20.1	28.7	/	FDA (2022), EMA (2022)

#### 2.3.1 Targeted Therapy plus Immunotherapy

Combining ICIs with targeted therapies has a strong scientific basis. For example, anti‐VEGF receptor angiogenesis monoclonal antibodies can alleviate local immunosuppressive effects of VEGF signaling and promote T cell infiltration [82]. Additionally, TKIs with anti‐angiogenic activity and various other kinase targets can potentially modulate the TIME in different ways, enhancing the response to ICIs [83]. Multiple studies have shown that combining immunotherapy with targeted therapy can achieve synergistic antitumor effects, making this treatment approach a hot topic in HCC clinical research.

##### 2.3.1.1 Atezolizumab plus Bevacizumab

Atezolizumab is a humanized anti‐PD‐L1 monoclonal antibody that binds to PD‐L1 receptors on tumor cells, blocking the PD‐1/PD‐L1 pathway. In 2020, the FDA approved atezolizumab combined with bevacizumab for patients with unresectable HCC who have not received prior systemic therapy, making it a new first‐line treatment option for advanced HCC [18]. A phase Ib clinical trial GO30140 (NCT02715531) found that atezolizumab combined with bevacizumab showed considerable clinical efficacy and good safety [84]. Subsequently, an open‐label phase III trial IMbrave150 (NCT03434379) confirmed that in advanced HCC, atezolizumab combined with bevacizumab resulted in longer mOS (19.2 months vs. 13.4 months) and longer PFS (6.9 months vs. 4.3 months) compared to sorafenib [85]. Although atezolizumab combined with bevacizumab is more effective, its high cost makes it a less cost‐effective choice as a first‐line treatment [86].

##### 2.3.1.2 Pembrolizumab plus Lenvatinib

At the 2018 American Society of Clinical Oncology (ASCO) meeting, the combination of pembrolizumab and lenvatinib debuted, showing a DCR of 100%. Among treatment‐naive patients, one achieved CR, the expansion cohort (treatment‐naive) had an ORR of 35%, the dose‐escalation cohort had an efficacy rate of 66.7%, and the DCR was also an impressive 100%. HBV patients also benefited, with durable efficacy and an mPFS of 9.69 months [87]. The FDA recognized pembrolizumab combined with lenvatinib as a breakthrough therapy for HCC. However, a 2023 phase III clinical trial LEAP‐002 (NCT03713593) showed that while pembrolizumab combined with lenvatinib as a first‐line treatment for advanced HCC exhibited good clinical activity with an mOS of 21.2 months and an mPFS of 8.2 months, both higher than the lenvatinib plus placebo group, the primary endpoints did not meet the preset targets, showing no significant statistical difference [88].

##### 2.3.1.3 Camrelizumab Plus Rivoceranib

At the 2022 European Society for Medical Oncology (ESMO) conference, the international phase III clinical trial CARES‐310 (NCT03764293), led by Chinese researchers, announced that the combination of camrelizumab and rivoceranib achieved both study endpoints, with OS and PFS reaching preset targets [89]. Results showed that the camrelizumab and rivoceranib combination treatment group had an mOS of 22.1 months, significantly benefiting compared to the sorafenib group (15.2 months), with an ORR of 25.4%, DCR of 78.3%, a short mTTR of only 1.9 months, and an mDOR of 14.8 months, demonstrating quick efficacy and long‐lasting remission [90]. Among the currently approved first‐line treatments for advanced HCC, the combination of camrelizumab and rivoceranib has set a new high for OS, offering significant survival benefits for HCC patients.

##### 2.3.1.4 Nivolumab plus Cabozantinib

Based on the series of studies on nivolumab, in cohort 6 of a phase I/II clinical trial CheckMate‐040 (NCT01658878), researchers tested nivolumab combined with cabozantinib or without ipilimumab for advanced HCC. Results showed that the dual‐drug group had improved mDOR (8.3 months vs. not reached) and mPFS (5.1 months vs. 4.3 months) compared to the triple‐drug group, but was weaker in ORR (17% vs. 29%) and mOS (20.2 months vs. 22.1 months) [91]. Thus, the triple‐drug group had higher and more durable response rates, but also showed more common grade 3–4 TRAEs than the dual‐drug group [60], necessitating more samples and clinical trials to verify the combination results.

#### 2.3.2 Dual Immunotherapy

Both PD‐1 and CTLA‐4 can inhibit downstream signaling of TCRs on CD8+ T cells, but through different mechanisms. CTLA‐4 competitively binds to B7, blocking the second signal (CD28‐B7 co‐stimulatory signal) required for T cell activation, directly inhibiting T cell activation [72]. PD‐1 is mainly expressed on activated T cells and, by interacting with PD‐L1, attenuates TCR/MHC and CD28/B7 stimulation signals, leading to downstream pathway dephosphorylation, preventing further T cell activation mediated by antigens [58, 59]. Therefore, combining anti‐PD‐1/PD‐L1 and anti‐CTLA‐4 antibodies can relieve immunosuppressive effects at different stages of T cell immune responses [92]. In clinical cohorts treating advanced liver disease, published data on dual immune combination therapies have also shown significant clinical improvement and long‐term survival benefits.

##### 2.3.2.1 Nivolumab Plus Ipilimumab

In 2020, the FDA approved the combination of nivolumab and ipilimumab for patients with HCC who had previously received sorafenib, making it the first approved dual immunotherapy regimen worldwide [93]. According to the recent phase III clinical trial CheckMate‐9DW (NCT04039607), the combination of nivolumab and ipilimumab achieved an mOS of 23.7 months, an ORR of 36%, with 7% of patients achieving CR, and an mDOR of 30.4 months [94]. These results, along with previous studies, indicate that the nivolumab and ipilimumab combination regimen has excellent potential in improving the survival of patients with advanced HCC, offering higher ORR and sustained responses with manageable safety profiles [95]. These results may support this combination as a potential new first‐line standard treatment for advanced HCC.

##### 2.3.2.2 Tremelimumab Plus Durvalumab

The combination of tremelimumab and durvalumab, known as the STRIDE regimen, is the first dual immunotherapy regimen approved for first‐line treatment of HCC [77]. The success of the STRIDE regimen is based on results from the phase III clinical trial HIMALAYA (NCT03298451). The STRIDE regimen showed significant differences in mOS (16.4 months vs. 13.8 months) and ORR (20.1% vs. 5.1%) compared to the sorafenib group. Moreover, in terms of safety, the STRIDE regimen group did not reveal any new safety signals, with lower rates of grade 3/4 TRAEs compared to the sorafenib group (25.8% vs. 36.9%), and no increased risks of severe hepatotoxicity and bleeding [96]. The STRIDE regimen utilizes the unique combination potential of CTLA‐4 and PD‐L1 inhibitors to enhance cancer immune response significantly, improving OS, making it a direction worth further exploration [97].

## 3 Current Challenges and Evolving Strategies

The rapid evolution of systemic therapy for advanced HCC, while transformative, has unveiled a new set of complex challenges that define the present therapeutic frontier. Despite the improved survival offered by targeted and immunotherapeutic agents, their efficacy is ultimately constrained by the dual hurdles of primary and acquired resistance. The clinical application of these therapies is further complicated by a critical lack of predictive biomarkers, variable and sometimes severe toxicity profiles, and significant economic burdens that limit accessibility. In response, the field is actively exploring novel strategies to navigate disease progression, including optimizing sequential therapy after first‐line treatment failure, developing more potent triplet combinations, and ambitiously expanding the role of systemic therapy into neoadjuvant and adjuvant settings. Addressing these limitations and integrating these emerging strategies is paramount for advancing toward a more personalized and effective management paradigm for HCC.

### 3.1 Limitations of Current Therapies

The initial success of molecular targeted agents and immune checkpoint inhibitors has revealed a more complex clinical reality, characterized by the inevitable emergence of resistance, a lack of tools for patient stratification, and substantial clinical and socioeconomic burdens. These challenges collectively undermine the long‐term management of the disease, highlighting that the present arsenal, while advanced, remains a stepping stone rather than a final solution.

#### 3.1.1 Primary and Acquired Resistance

The development of resistance, both primary and acquired, remains a formidable barrier to the long‐term efficacy of systemic therapies in HCC. Approximately 15% to 40% of patients experience disease progression during initial therapy, which is referred to as primary resistance. A significant contributor is tumor heterogeneity. Studies utilizing large‐scale patient‐derived organoid (PDO) biobanks have revealed substantial intra‐tumor heterogeneity, which is a key determinant of initial treatment failure. For instance, distinct subclones within a tumor may pre‐exist with molecular features that confer insensitivity to TKIs like lenvatinib, often mediated through pathways such as JNK and β‐catenin signaling [98].

Acquired resistance emerges after an initial period of clinical benefit, often driven by the clonal evolution of tumor cells toward a low immunogenicity phenotype under the selective pressure of therapy. Mechanisms involve the activation of alternative signaling pathways, such as the CARM1‐TRIM47‐SNAI1 axis, where the stabilization of TRIM47 promotes EMT and metastasis, providing a route for HCC to evade treatment [99]. Similarly, feedback activation of the EGFR pathway has been identified as a key mechanism of resistance to Lenvatinib [100]. Metabolic reprogramming and immune evasion are equally critical factors in drug resistance. Tumor cells can reshape metabolic mechanisms to resist treatment. The gap junction protein GJB2 has been shown to activate the glycolytic pathway, which not only promotes HCC proliferation and metastasis but also creates an immunosuppressive TME [101]. Meanwhile, CRKL protein can induce infiltration of tumor‐associated neutrophils via β‐catenin, VEGF‐α, and CXCL1. These alterations in the TME lead to immune evasion, diminishing the efficacy of PD‐1 blockade therapy [102].

#### 3.1.2 The Lack of Predictive Biomarkers

The advancement of HCC systemic therapy is significantly hampered by the critical shortage of validated predictive biomarkers, rendering treatment selection largely empirical and impeding personalized medicine. The most prominent exception is AFP, which guides the use of ramucirumab for patients with AFP ≥400 ng/mL, as demonstrated in the REACH‐2 trial [52]. Beyond this, robust biomarkers for first‐line therapies are urgently needed.

Research has identified potential biomarkers linked to drug susceptibility. For sorafenib, mutations in the PI3K‐AKT‐mTOR pathway are associated with poorer response, while VEGFA amplification and ACSL4 expression may predict better outcomes [103, 104, 105]. For lenvatinib, candidates include FGF19, ST6GAL1, VEGF, and ANG2 [106, 107]. For immune checkpoint inhibitors, a pre‐existing inflamed tumor microenvironment, characterized by high CD8+ tumor‐infiltrating lymphocytes and inflammatory gene signatures (e.g., CD8A, CD274, LAG3), correlates with improved survival with nivolumab and atezolizumab plus bevacizumab [108]. Furthermore, a lower T‐regulatory/T‐effector cell gene signature and TERT promoter mutations are associated with better outcomes in patients receiving atezolizumab plus bevacizumab [109]. However, tumor heterogeneity and the dynamic tumor microenvironment pose substantial challenges to the development of a single, universal biomarker. Most candidates remain investigational, underscoring a pressing need for their standardized validation and clinical integration to optimize patient stratification and treatment efficacy.

#### 3.1.3 Treatment‐Related Toxicity

Treatment‐related toxicity is a major limitation of current systemic therapies for HCC, often leading to dose reductions, treatment discontinuation, and compromised quality of life, which can ultimately curtail their clinical benefits. The toxicity profiles vary significantly between drug classes and combination regimens. TKIs are associated with predictable class‐effects. Sorafenib commonly causes diarrhea, fatigue, and HFSR [46]. Regorafenib and cabozantinib also show high rates of HFSR, hypertension, and fatigue [40]. Lenvatinib frequently leads to hypertension, diarrhea, decreased appetite, and weight loss [35]. Furthermore, patients with compromised liver function, such as Child‐Pugh B class, are more susceptible to hepatic deterioration and severe adverse events from TKIs like sorafenib and lenvatinib [110], whereas ICIs may be relatively better tolerated in this population from a hepatic metabolism perspective [60].

ICIs introduce a distinct spectrum of irAEs caused by T‐cell activation against healthy tissues. These can affect any organ, with common manifestations including rash, colitis, hepatitis, and endocrinopathies. The combination of nivolumab and ipilimumab, while effective, induces grade 3/4 irAEs in a significant proportion of patients, often requiring systemic corticosteroids [95].

### 3.2 Salvage and Pioneering Strategies

The dilemma of sequential therapy arises from the lack of a standardized standard of care (SoC) following progression on first‐line targeted‐immunotherapy combinations. Current guidelines, largely based on data from the TKI era, provide limited guidance for post‐immunotherapy scenarios. Clinical evidence for second‐line regimens remains sparse and often derives from small, retrospective studies. We presently face two critical challenges: managing patients after failure of targeted‐immunotherapy and enhancing the efficacy of first‐line treatment.

#### 3.2.1 The Challenge of Sequential Therapy

There is a notable scarcity of robust phase III evidence specifically guiding treatment after the failure of first‐line ICIs or ICI‐based combinations. For instance, a phase II study presented at ASCO 2024 evaluating regorafenib plus pembrolizumab after ICI progression showed limited benefit, particularly in patients who had previously received combination immunotherapy [111]. At the same time, the issue of “cross‐resistance” exists, and it is still unclear whether resistance to one ICI regimen will lead to resistance to other ICIs or TKIs. As an illustration, the efficacy of switching to CTLA‐4 inhibitor regimens—such as nivolumab plus ipilimumab—following failure of PD‐1/L1 blockade remains unconfirmed [112], while the therapeutic value of TKI use after ICI progression requires further validation.

However, as a salvage therapy, switching to an alternative TKI is commonly employed for patients with disease progression following ICI‐based combination therapy [8]. Specifically, regorafenib, cabozantinib, or lenvatinib—provided they were not administered in prior treatment lines—are often considered as potential salvage options, despite the lack of a fully established evidence base. To address this critical knowledge gap and standardize the second‐line therapeutic approach for such patients, the global phase III clinical trial IMbrave251 (NCT04770896) has been designed to directly compare the therapeutic outcomes of sorafenib/lenvatinib versus atezolizumab combined with sorafenib/lenvatinib regimen in patients whose disease progressed after initial treatment with atezolizumab plus bevacizumab. This trial is anticipated to generate level 1 evidence to guide clinical decision‐making for this patient population.

#### 3.2.2 Novel Combination Therapies

The failure of first‐line therapy has spurred the development of more intensive and innovative treatment strategies. Moving beyond doublet regimens, the field is increasingly exploring novel combinations that involve triple or even multi‐drug therapies, as well as the sophisticated integration of systemic agents with locoregional and surgical modalities.

Dual immunotherapy plus targeted therapy represents a viable approach. The potent yet highly toxic combination of nivolumab and ipilimumab has been approved as a second‐line treatment. If combined with TKIs, it may yield longer‐term benefits. Moreover, combining ICIs with multiple TKIs also holds potential. Building on the success of TKI‐ICI combinations like camrelizumab plus rivoceranib, adding a third TKI targeting a distinct, non‐overlapping pathway, such as VEGFR or MET, may yield greater efficiency. Additionally, a triple‐agent strategy in advanced biliary tract cancer (IMbrave151, NCT04677504) offers valuable insights. The combination of atezolizumab with bevacizumab and chemotherapy regimen demonstrated an objective response rate of 76.7% in a retrospective analysis, with manageable safety [113]. However, while combination therapies enhance efficacy, they also increase the risk of adverse events. Managing these toxicities, particularly irAEs and overlapping toxicities induced by anti‐angiogenic agents, is a critical component of patient care.

The synergistic “local plus systemic” treatment approach represents a superior strategy for HCC. Systemic therapy combined with TACE/HAIC demonstrates biological rationality. TACE/HAIC induces tumor necrosis, releasing tumor‐associated antigens and damage‐associated molecular patterns to elicit an in‐situ vaccine effect, which may reverse ICI resistance. Key results of the phase III clinical trial TALENTACE (NCT04712643), presented at ESMO GI 2025, validated this strategy. The combination of atezolizumab plus bevacizumab with TACE showed superior efficacy and safety versus TACE. The mPFS was 11.30 months vs. 7.03 months, and the ORR was 81.3% vs. 66.7% [114]. Simultaneously, a groundbreaking approach involves quadruplet therapy: combining intraluminal brachytherapy (I‐125 seed strand), portal vein stenting, TACE, and systemic therapy (lenvatinib plus anti‐PD‐1 antibody). A propensity‐score analysis showed that this intensive strategy led to superior intrahepatic tumor control (55.3% vs. 17.5%), longer mOS (17.7 vs. 12.0 months), and longer PFS (17.0 vs. 8.0 months) compared to a regimen without immunotherapy [115]. Remarkably, this approach successfully downstaged some patients to undergo curative‐intent liver transplantation or surgical resection.

## 4 Summary and Outlook

HCC has entered a new era defined by the combination systemic therapies and the pursuit of individualized treatment strategies [15]. Despite these advancements, significant clinical challenges persist, primarily concerning the prediction of responses to first‐line regimens and the formulation of effective sequential therapies upon disease progression [3].

Systemic therapy can prolong survival in some patients with advanced hepatocellular carcinoma [21]. However, drug resistance and side effects limit its efficacy [116]. When first‐line therapy fails, the subsequent strategy must be tailored to the pattern of progression. For intrahepatic localized progression, a rational approach involves consolidating the existing systemic regimen with local interventions such as TACE, HAIC, or stereotactic body radiotherapy [8]. This multimodal strategy can effectively control dominant lesions and may potentially reverse local immunosuppression. In cases of widespread progression, a switch in systemic therapy is warranted. Following progression on a regimen like atezolizumab plus bevacizumab, second‐line options may include switching to a TKI or a dual‐immunotherapy combination [16]. Novel triple or quadruple therapies, which integrate TKIs, ICIs, and other modalities like I‐125 seed brachytherapy, are showing promise in clinical trials for challenging scenarios like portal vein tumor thrombus, offering new avenues to overcome multidrug resistance [115].

A critical hurdle is the current lack of robust, universally accepted biomarkers for predicting outcomes to first‐line combinations [14]. Emerging evidence suggests that molecular and etiological factors play a pivotal role. For instance, mutations in the Wnt/β‐catenin pathway are associated with primary resistance to immunotherapy, potentially by fostering an immune‐excluded tumor microenvironment [117]. In contrast, baseline AFP levels and inflammatory gene signatures within the TME may help identify patients more likely to respond to combinations such as atezolizumab plus bevacizumab. Etiology further stratifies responses, HBV‐associated HCC typically demonstrates superior outcomes with combined immunotherapy and anti‐angiogenic agents, whereas non‐viral HCC, particularly with Wnt/β‐catenin activation, often exhibits limited benefit [87]. These observations underscore the necessity of biomarker‐driven selection among first‐line options, which currently include ICI‐based combinations with either anti‐angiogenic agents or other ICIs [118].

Beyond the palliative setting, systemic therapy is expanding into curative‐intent domains [119]. Targeted‐immunotherapy combinations serve as effective conversion therapies, successfully downstaging a subset of initially unresectable HCCs to resectable status [120]. Their role as a bridge to liver transplantation and in the adjuvant setting post‐resection is also being actively investigated, highlighting a paradigm shift toward a more integrated and ambitious treatment landscape [121].

In conclusion, the future of personalized HCC therapy hinges on the integration of multi‐omics biomarkers—including ctDNA, tumor mutational burden, and TME profiling—to guide clinical decision‐making. A deeper understanding of resistance mechanisms and the development of etiology‐ and genotype‐specific therapeutic sequences will be paramount to optimizing the entire patient journey and ultimately improving overall survival.

## Author Contributions

Yongxin Yu and Yulang Jiang conceptualized and developed the design of the article, interpreted relevant literature, and wrote the original manuscript. Yipeng Yang and Christian Glandorff revised the manuscript. Wenzheng Fang proofread the manuscript. Mingyu Sun was responsible for conceptualization, writing—review & editing, funding acquisition, and validation. All authors have read and approved the final.

## Funding

This work was supported by the Major Project of Shanghai Municipal S and T Commission (No. 19401972300), Shandong Province Key R&D Program (Major Science and Technology Innovation Project, 2021CXGC010509), Key Laboratory of Chronic Deficiency Liver Disease of the State Administration of Traditional Chinese Medicine of the People's Republic of China (20DZ2272200), Shanghai Key Specialty of Traditional Chinese Clinical Medicine (shslczdzk01201), Mechanism Study of Traditional Chinese Medicine Formulations Suppressing Hepatocellular Carcinoma by Regulating Ubiquitination Processes and the Tumor Microenvironment (2025‐22), Exploring Cellular and Molecular Mechanisms of Gastric Cancer Development Using Artificial Intelligence‐Based Deep Learning Techniques (2024‐02).

## Ethics Statement

Ethical approval is not applicable to this review article because the study exclusively utilized aggregated data from previously published clinical trials. All original trials included in our analysis were conducted in compliance with ethical standards and had obtained appropriate institutional ethics committee approvals.

## Conflicts of Interest

The authors declare that they have no conflicts of interest.

## Data Availability

The data generated in the present study may be requested from the corresponding author.
